# Bond Performance of Steel Bar and Fly Ash-Based Geopolymer Concrete in Beam End Tests

**DOI:** 10.3390/polym14102012

**Published:** 2022-05-14

**Authors:** Yifei Cui, Shihao Qu, Jiuwen Bao, Peng Zhang

**Affiliations:** 1 Center for Durability & Sustainability Studies of Shandong Province, School of Civil Engineering, Qingdao University of Technology, Qingdao 266033, China; qushihao2022@163.com (S.Q.); baojiuwen@qut.edu.cn (J.B.); 2 School of Engineering and Information Technology, University of New South Wales, Canberra, ACT 2612, Australia

**Keywords:** geopolymer concrete, reinforced concrete, bond–slip, steel bar

## Abstract

This paper presents a comprehensive investigation of the bond characteristics of steel bar reinforced geopolymer concrete (GPC). The ASTM A944 beam end tests were conducted on GPC beams reinforced with plain or ribbed bars. The bond–slip curves and the bond strength of GPC beams were obtained. The relationship between the bond stress and relative slip in plain and ribbed bar reinforced GPC has been represented by empirical formulae. The bond testing results were compared with those of corresponding ordinary Portland cement concrete (OPC) using statistical hypothesis tests. The results of hypothesis testing showed that GPC was significantly superior to OPC in terms of bond capability with plain bars and bond stiffness with ribbed bars. The statistical analysis indicated that the bond–slip relations derived for OPC are inapplicable to GPC; thus, new bond–slip relations are suggested to estimate the development of bond stress and relative slip between GPC and steel bars.

## 1. Introduction

Ordinary Portland cement has been criticized for its high energy consumption in its production process [1] and low durability in its service life [2]. For decades, researchers have been searching for low-energy consumption sustainable binders [3]. Geopolymers are potential alternative binders because of their total environmental friendliness, outstanding mechanical properties and acceptable processing costs [4]. The mechanical properties of geopolymer concrete are comparable to traditional concrete, while in severe environments, such as acid [5], sulphate attack [6], chloride [7] and elevated temperature [8], geopolymer binder provides superior residual strength. Although the general cost for producing geopolymer binder is similar to that of Portland cement [9], geopolymers present almost 25% less carbon emission [10].

Geopolymers are sustainable inorganic polymers that have internal chemical compositions quite similar to natural zeolitic materials despite their amorphous microstructures [11]. The formation of geopolymers is a series of complex reactions which could be simplified as the alkali-activation and polymerisation. The typical micro composition of geopolymer gel is composed of tetrahedral (4-coordinated) Al and Si atoms framework and tetrahedral AlO_4_ groups that are charge-balanced by nonframework sodium ions [12]. There are various materials that can be used to synthesize geopolymers. Natural minerals and industrial by-products that concentrate aluminosilicates, such as metakaolin, red mud and fly ash, have all been found to be suitable sources for geopolymer production [13].

Among them, fly ash is one of the most popular raw materials for the production of geopolymers, an important by-product of the power generation industry. Fly ash-based geopolymers are synthesized by activated fly ash with alkaline activators such as KOH, NaOH, other alkali metal hydroxides, carbonates and silicates [14]. Alkaline compound transforms the glassy structures of fly ash into compact well-cemented composites [12,15].

The chemical structure of alkaline-activated fly ash provides many outstanding engineering characteristics. For instance, the fly ash microspheres act as fillers of voids in concrete and thus improve the workability of fresh concrete. As a consequence, dense concrete is produced with better tensile resistance [16]. Previous research has reported that geopolymer concrete (GPC) outperforms ordinary Portland cement concrete (OPC) with respect to compressive strength [17], chemical resistance [18] and bonding with reinforcing bars [19].

In OPC, hydrated cement works as a binder of the fine and coarse aggregates that bond the concrete matrix together. In GPC the binder is provided by geopolymerization, which is the process of combining aluminate and silicate molecules dissolved from fly ash into a three-dimensional network [20]. Binders also bond the reinforcing steel and the surrounding concrete. Therefore, as the binder changes from Portland cement to geopolymer, the bond characteristics between them change.

The bond is the most important interaction between the bar and concrete. Bond strength can be attributed to three different contributors [21]: (1) chemical adhesion between the binder and the steel bar surface; (2) the friction of small, dislodged particles between the bar and the surrounding concrete; and (3) wedging action between concrete and ribs. Among them, the last only occurs in ribbed bar reinforced concrete. When tensile force is applied to the ribbed bar, the steel ribs push the concrete in front of it, and the surrounding concrete reacts to the thrust and begins transmitting force within the concrete [22]. Compared with this mechanical interaction, friction and chemical adhesion forces are secondary and decrease quickly as the reinforcing bars start to slip [23]. Therefore, the bond strength of ribbed bars is significantly higher than that of plain bars in bond strength tests.

Previous studies on the bonding of reinforced GPC components have mainly focused on experimental investigations [24]. Sarker used the beam end pull out test [25,26] to investigate the bond strengths of GPC and reported that the bond strength of GPC is higher than that of OPC in pull out tests. Consistent with Sarker’s results, Fernández-Jiménez [27] and Zhang [28] found the bond strength of ribbed bar reinforced GPC is higher than that of OPC in direct pull out tests. It turned out that the bond capability of GPC is considerably greater than that of OPC at both room and high temperatures. Sofi [29] reported that reinforced GPC requires less bond length than that recommended by standard design equations (AS 3600 [30], EC2 [31] and ACI-318 [32]). Castel and Foster [33] also agreed that the bond strength between steel bars and geopolymer concrete is higher than the bond strength of reinforcing steel bars embedded in OPC concrete. In addition to the bond strength, it has been found that the interfacial bond stress distribution in GPC is different from that in OPC [19].

The above studies have provided preliminary results of the bond performance of GPC. It has been agreed that not only bond strength but also other bond properties such as bond stiffness and bond stress distribution will differ significantly between GPC and OPC. However, the analytic formula used to predict the composite performance of reinforced OPC cannot be used for reinforced GPC. There is insufficient proof in existing bond studies on qualitative differences that may encourage the publication of new GPC bond standards. To contribute to the work of GPC bonding criteria and to investigate the technical potential of GPC, this study uses statistical hypothesis testing to define significant differences in bond behaviour between GPC and OPC. Specific correlations were also proposed to predict the bond–slip behaviour between GPC and reinforcements.

## 2. Experimental Programme

The experimental work consists of the investigation of the bond–slip behaviour of GPC in pull out tests and the comparison between GPC and identical OPC concrete specimens. A class F fly ash-based GPC mix and a corresponding OPC mix with corresponding compressive strengths were prepared and the OPC and GPC specimens were subjected to ASTM A944 [34] bond testing.

### 2.1. Materials

#### 2.1.1. Concrete

The tests used ASTM Class F fly ash [35] as the raw material for geopolymers. Two batches of fly ash referred to as ‘CFA1’ and ‘CFA2’, respectively, were used and the XRF and LOI results are listed in Table 1.

Sodium hydroxide and water glass were used as alkali activators. The sodium hydroxide solution had a concentration of 12 M and was prepared by dissolving commercial 98% pure flakes (supplied by Redox Pty Ltd., Centriair Pty Ltd., Sydney, Australia) in water. Water glass is laboratory grade D sodium silicate solution with a SiO_2_/Na_2_O ratio between 1.95 and 2.05 and was procured from IMCD Australia Limited. The coarse aggregates sized 14 mm, 10 mm and 7 mm and river sand were prepared with saturated surface dry (SSD) conditions before mixing. A superplasticizer (CENTROXTM^®^ HWR, Sydney, Australia) and a viscosity modifier (CENTROXTM^®^ VM, Centriair Pty Ltd.,Sydney, Australia) were applied at a dosage of 900 mL per 100 kg fly ash, respectively.

The control group used Portland cement from CEMENT AUSTRALIA^®^, which complies with the Australian Standard AS 3972 [36] requirements for Type GP cement.

#### 2.1.2. Mix Design

The OPC mix was designed according to the British method [37], while the GPC mix used was consistent with early research [38]. The mix proportions are given in Table 2.

The two mix designs shown in Table 2 are based on SSD condition aggregates with a targeted compressive strength of 35 MPa.

#### 2.1.3. Steel Bars

The Australian normal ductility hot-rolled ribbed bars and plain bars from One Steel^TM^, Sydney, Australia were used in the present study. The diameter/nominal diameter of the plain/ribbed bars was 16 mm. All the bars were cleaned using sandpapers and alcohol wipes before casting. Samples of the steel bars were tested in the UNSW@ADFA laboratory to obtain the mechanical properties. Test results are given in Table 3.

### 2.2. Beam End Specimens

The ASTM A944 [34] beam end test was used in order to test the bond performance of reinforced members under similar stress states as those seen under service conditions.

**Figure 1:**
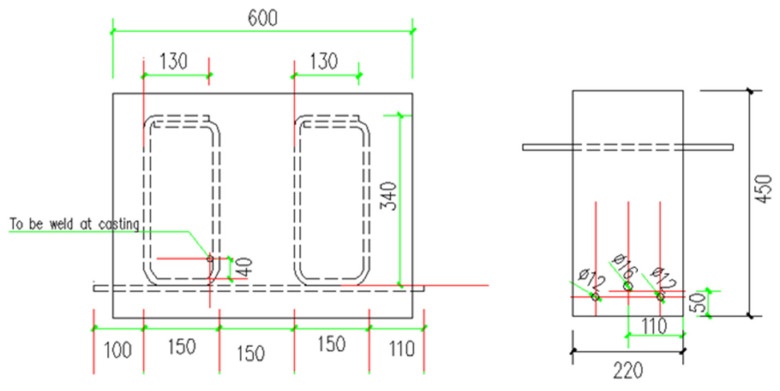
Schematic diagram of a beam end specimen.

In total, 12 reinforced GPC beams and 12 OPC beams were cast and subjected to beam end test. All the mixes were made in the UNSW@ADFA laboratory using a 120 L concrete mixer. The concrete cover (c) is 50 mm. The geometry of the specimens is shown in Figure 1.

### 2.3. Cast and Testing

The GPC samples were manufactured and cured according to procedures reported by earlier research [19,38,39]. The rust and dirt on the test bars were removed with sandpaper and cleaned with ethanol. The steel bars were placed in the bottom position before casting. Every beam was cast with several standard 100 mm × 200 mm cylinders to test the compressive strength of the concrete. The beam and cylinders were placed in the environmental control room (ER, 20 ± 1 °C, 50% humidity) for 24 h. The GPC specimens were then moved to an insulated chamber at 80 °C for 24 h and left in the laboratory for ambient curing until the time of testing. The OPC specimens were moved to a moist room (ER, 20 ± 1 °C, 100% humidity) on the day following casting until the day of testing. It has been reported that GPC can reach approximately 90% compressive strength within 7 days of heat curing [38,39], thus GPC compressive strength and beam end testing were conducted on day 7. The mean value of the 28-day compressive strength of the OPC cylinders is 36.84 MPa, while the mean value of the 7-day strength of the GPC is 35.4 MPa.

**Figure 2:**
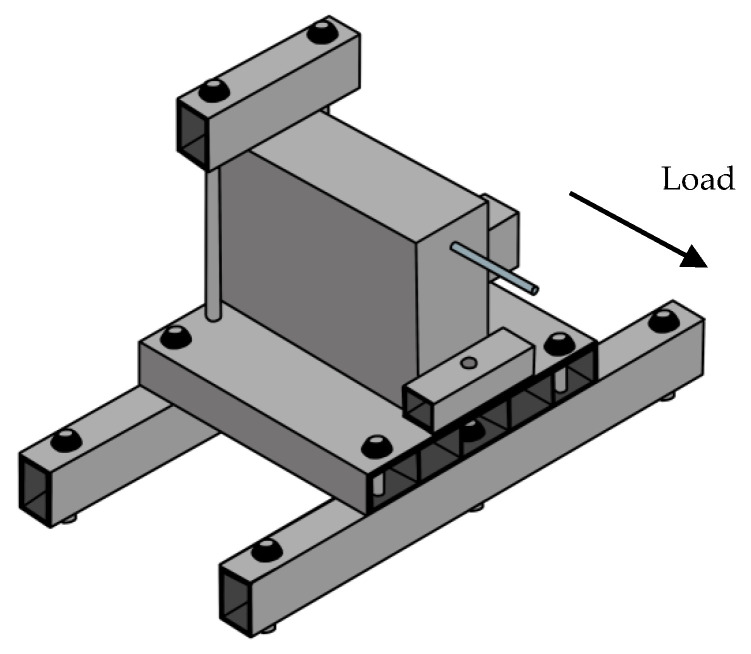
Sketch of the beam end pull out test.

The test samples were divided into two series; 12 plain bar reinforced samples and 12 ribbed bar reinforced samples. Before the tests, beams were settled on the test rig on the strong floor by crane (Figure 2). The requirements of ASTM test standard ASTM A944 [34] were followed throughout testing.

The specimens were loaded using an INSTRON^®^ hydraulic (MEAS, Hampton, VA, USA) actuator with a loading rate of 2 kN/min for plain bars and 12 kN/min for ribbed bars. The test bar was pulled out by the SHIMADZU^@^ MWG-100kNA (SHIMADZU, Kyoto, Japan) wedge grip under the designed loading rate during the test. The relative slip at the free end was measured by a pair of SCHAEVITZ^®^ 050-HR (inch/5000) LVDTs (MEAS, Hampton, VA, USA), and a pair of MICRO-MEASUREMENTS^®^HS25 (25 mm) LVDTs (Vishay Precision Group, Inc., Wendell, NC, USA) at the loaded end of the bar. In the test procedure, the relative slippage between steel and concrete was continuously read by four linear variable differential transformers (LVDTs). The load, slip and strain were collected at a rate of 10 points per second.

## 3. Results and Discussion

The observed failure phenomenon of the specimens and the data recorded by the acquisition system are studied in this section. The load–slip data were analysed, and the average load–slip curves obtained for the GPC and OPC samples of each series were determined. In addition, strain gauges were added to half of the samples and the steel load-strain curves and bond stress distribution were illustrated in another work [19].

### 3.1. Failure Type

The stress conditions between steel bars and concrete are a series of complicated stress redistribution. The failure of specimens is due to the failure of the concrete or the loss of the bond.

In this study, all the plain bar reinforced beams failed by pulling out of the steel. For the plain bar, friction and adhesion formed the bond forces on the interfacial area, hence the bond strength is determined by the interfacial condition. When the bond between the plain bar and the surrounding concrete is incapable of resisting the pull out load, the slip will occur on the steel-concrete interface, and the concrete will experience pull out failure.

In the case of the ribbed bar, the effect of chemical adhesion is considerably smaller than that of the mechanical interlock forces and only occurs at the beginning. The ribs on the bar bite into the surrounding concrete. As relative slip increases, the chemical adhesion disappeared and the friction decreased, leaving the forces at the contact faces as the principal bond supplier. With the increase in pull out load, the concrete will fail in splitting when the stress in concrete reaches the limitation and cracks reach the surface of the concrete [40,41]. In this study, all the ribbed bar reinforced beams failed by splitting the concrete.

#### 3.1.1. Plain Bar

**Figure 3:**
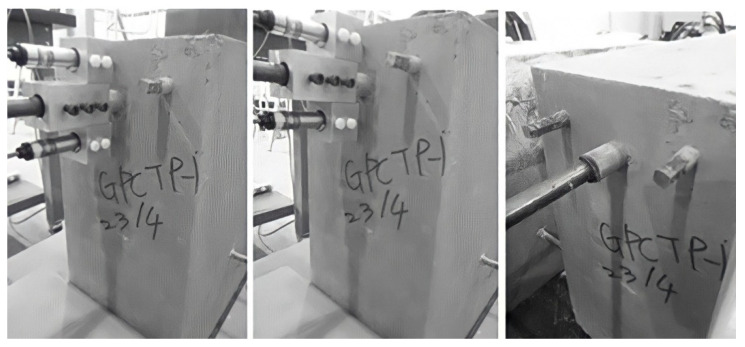
Front face images of plain bar reinforced GPC specimens: photos taken before (**left**), during (**middle**) and after (**right**) the beam end test.

**Figure 4:**
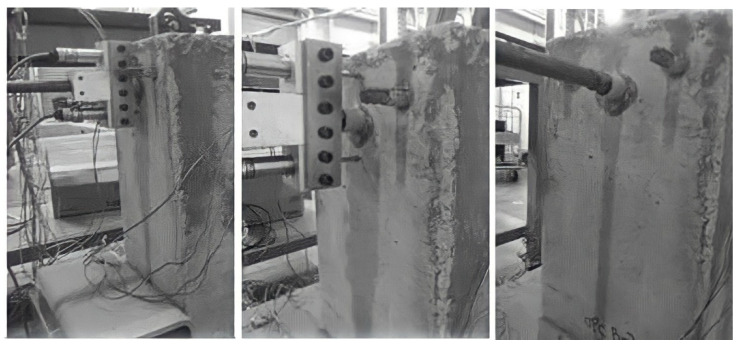
Front face images of the beam end test of plain bar reinforced OPC specimens: photos that were taken before (**left**), during (**middle**) and after (**right**) the beam end test.

The photo of the testing of the plain bar reinforced GPC and OPC concrete beam end specimens are shown in Figure 3 and Figure 4, respectively.

The plain bar reinforced GPC and OPC specimens had no visible cracks on the surface of the concrete matrix after the bars were pulled out. It seems that the inner shear cracks caused by debonding between the concrete and steel during the pull out procedure were restricted to the interfacial area and did not reach the surface of the matrix. Since no tensile cracking is likely to occur along the plain bar, sudden splitting failure is unlikely to develop. Tests were stopped manually when the value of slip reached the limit of the two free ends of the LVDTs.

#### 3.1.2. Ribbed Bar

The phenomenon of concrete splitting was observed in all the ribbed bar reinforced beam end specimens and was similar regardless of the concrete type. Splitting failure of the concrete matrix was sudden and occurred without any warning signs. It occurred without any external preliminary signs, as, before failure, no cracks were observed on the concrete surface, but at the moment of failure, cracks immediately reached the surface and split the matrix. Such brittle splitting failure of GPC specimens has also been observed by Sarker [42] and Sofi et al. [29].

The ribs on the bars were responsible for the occurrence of cracks. The bond force in ribbed bar reinforced concrete spread to the surrounding concrete and was no longer parallel to the central line of the pull out direction, as was observed in plain bar reinforced specimens. When subjected to pull out load, the resultant force exerted by the ribs on the concrete is inclined at an angle to the axis of the bar. The radial component of this resultant force caused the splitting of the surrounding concrete [43].

The test results demonstrated that the ribbed GPC and ribbed OPC both failed by splitting the concrete and showed similar splitting cracks after failure. Both ribbed GPC and OPC failed with the brittle manner of splitting failure following tension stress. However, the splitting of GPC is more abrupt than that of OPC. Hydraulic cement gel is full of separated capillary and gel pores while the pores in the geopolymer are all connected together to some degree [12,15]. Therefore, once a crack reaches a pole in geopolymer gel, it will spread straight through all the pores faster and easier and thus split the concrete instantly.

**Figure 5:**
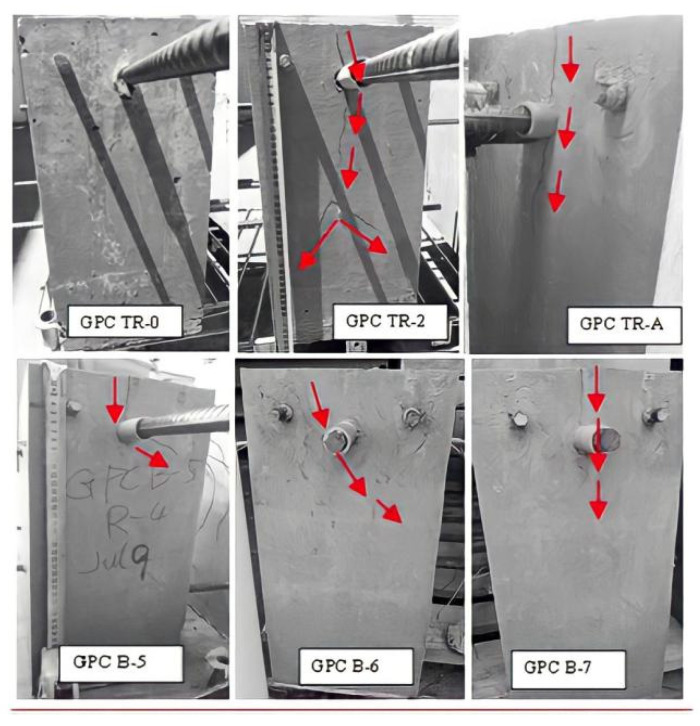
Examples of pull out face cracks in GPC beams (faint cracks are highlighted with red arrows).

**Figure 6:**
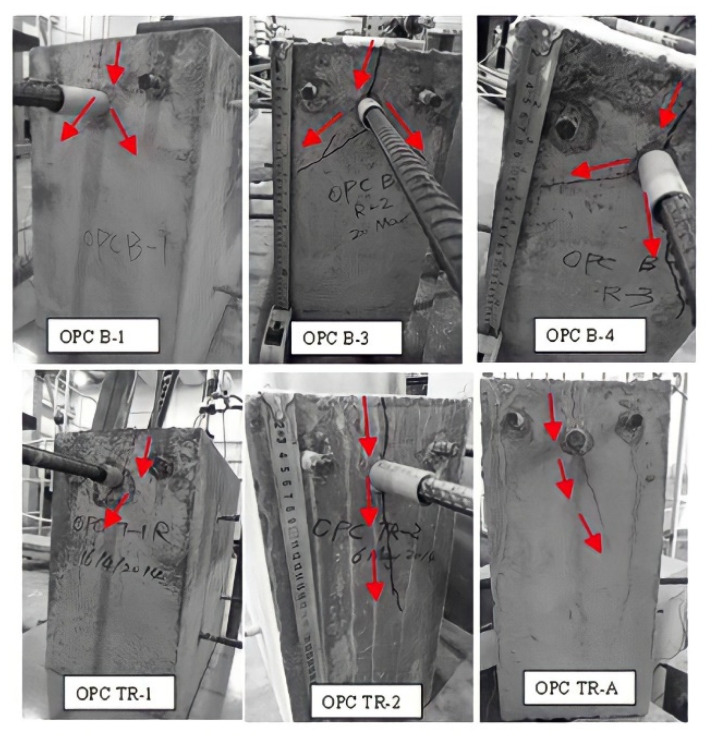
Examples of pull out face cracks in OPC beams (faint cracks are highlighted with red arrows).

The radial component dispersed by the ribs reaches the tensile strength, the concrete cover cracks and vertical crack can be observed running through the bonding area to the concrete surface [22,40]. Figure 5 and Figure 6 show the crack patterns on the pull out faces of the concrete matrix.

Figure 5 and Figure 6 illustrated that the cracking on the pull out faces of all specimens was similar regardless of concrete type. The main cracks were all perpendicular to the area with the least concrete cover. This is due to the failure mechanism of split failure do the decrease in tensile strength of concrete. The ribs convert the longitudinal load into a three-dimensional load that acts like hydraulic pressure in a concrete matrix. Splitting failures are caused by concrete material failures and work much more complicated than pull out failures. The OPC and GPC specimens were identical in geometry, reinforced bar, test schematic, loading rate, and concrete cover. Therefore, the splitting failure occurring in both the OPC and GPC specimens indicates the similar response of the two concretes to pull out load. The heterogeneous nature of concrete leads to anisotropic damage characteristics. In particular, the tensile strength of all types of concrete is much lower than the compressive strength. As mentioned above, split failure is determined by the tensile strength of the concrete. Therefore, similar cleavage cracks shown in GPC and OPC reflect similarly low resistance to tensile stress.

**Figure 7:**
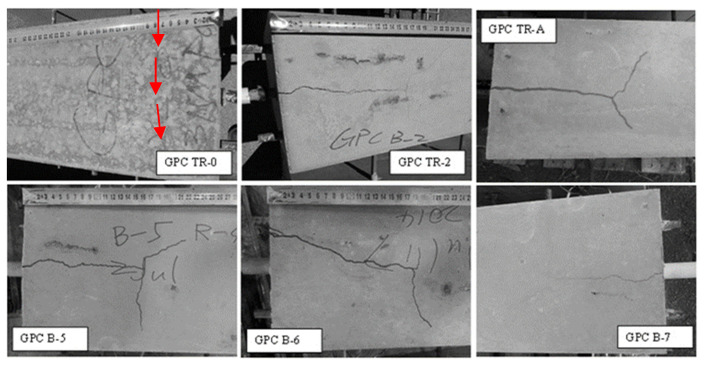
Examples of top face cracks in GPC beams (faint cracks are highlighted by red arrows).

**Figure 8:**
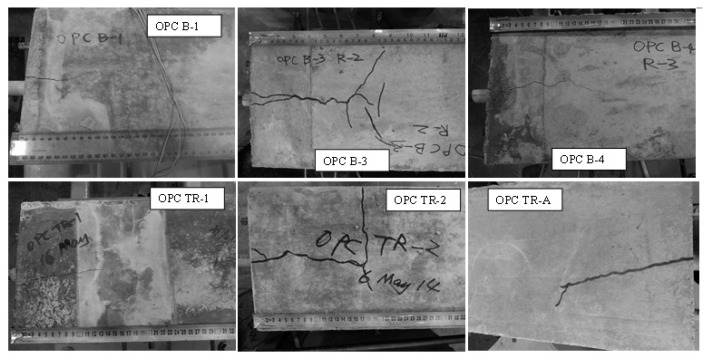
Examples of top face cracks on OPC beams.

Cracks seen on the pull out face of the beams are splitting cracks caused by the pull out load. On the top face, the stress conditions are much more complex, with those cracks caused by pull out and bending forces. Examples of cracks on the top faces are shown in Figure 7 and Figure 8.

It was observed that the splitting cracks that run through the pull out face to the top face were parallel to the steel bar, while flexural cracks were generated in the direction perpendicular to the pull out axis. The occurrence of flexural cracks illustrates that the beam end test is capable of simulating the flexural–tension stress experienced in service conditions.

For one beam end sample, GPC TR-0, no cracks were observed on its pull out face. This beam had a lower compressive strength (28.99 MPa) than the others. The matrix failed as the result of pull out and bending before the splitting of the concrete, with only a fine crack observed perpendicular to the pull out direction on the top face.

**Figure 9:**
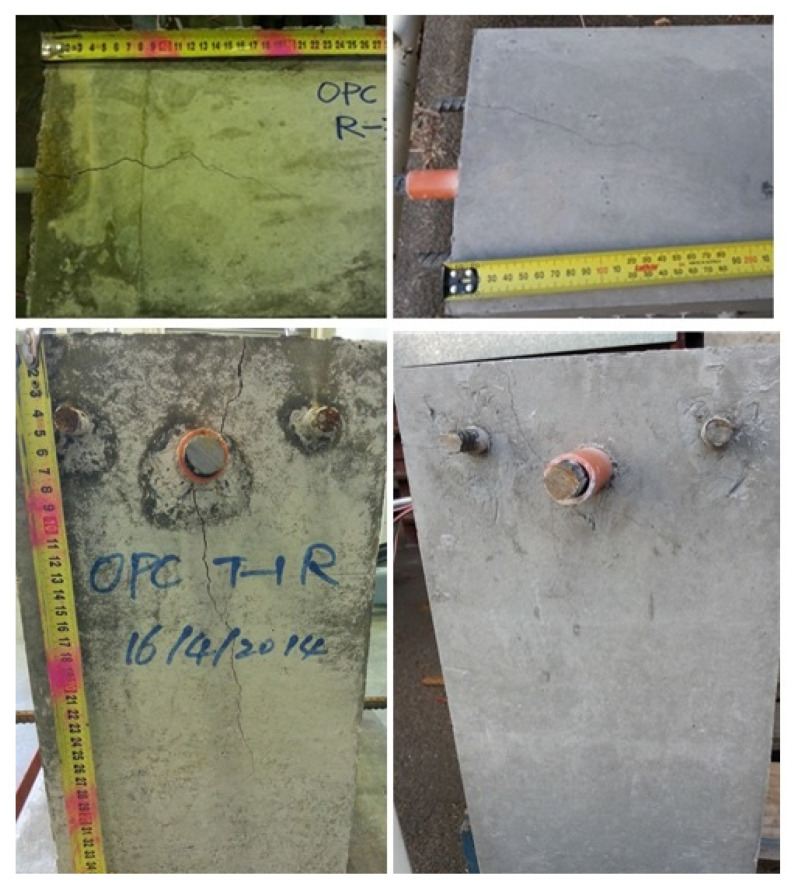
Comparison of the crack widths of OPC B-4 (left) and GPC B-6 (right).

Another noticeable point is that the widths of the cracks measured through a digital microscope at the GPC beams (typically 0.2–1 mm) were smaller than those in the OPC concrete beams (typically 0.5–2.5 mm). Additionally, the main cracks of OPC concrete were usually accompanied by tiny hairline cracks protruding out along or near their ends, while those of GPC were relatively ‘clean’, as shown in Figure 9.

To study the differences in crack morphology between GPC and OPC, a few tested beams were cut and the concrete on top of the reinforced bars was removed to explore the nature of the interaction at the steel–concrete interface.

**Figure 10:**
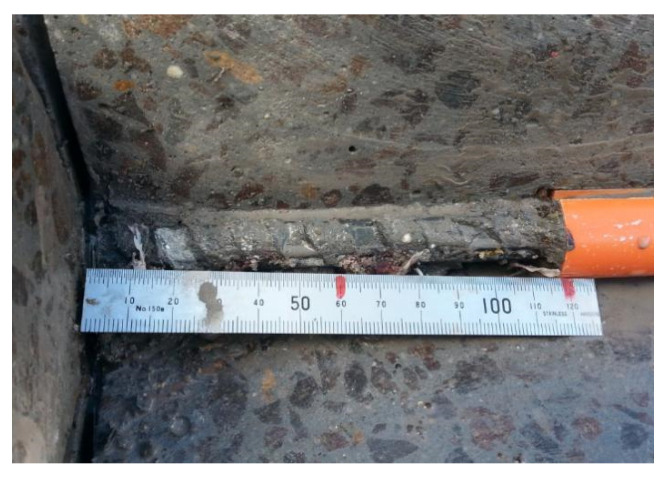
Steel-concrete interface on the GPC beam.

Figure 10 shows the GPC steel interface of beam GPC B-7. There have been no visible cracks or crushing observed on the concrete in front of the ribs. 

The binder was still firmly stuck to the ribbed bar after the pull out tests. It is clear that the strong chemical adhesion between geopolymer binder and steel let them resist the pull out load together and reduced the cracks and deboning around ribs. This phenomenon has also been observed by Chang [44]. The well bonded interfacial area explained the ‘clean’ cracks observed on the surface of GPC beams in Figure 7, Figure 8 and Figure 9. When the bar was pulled out from GPC beams, the binder in front of the ribs was still stuck to the steel and thus was sheared out from the surrounding concrete. This kind of failure spread toward the surface of the beam and left a relatively ‘clean’ main crack on the surface of the GPC beams.

**Figure 11:**
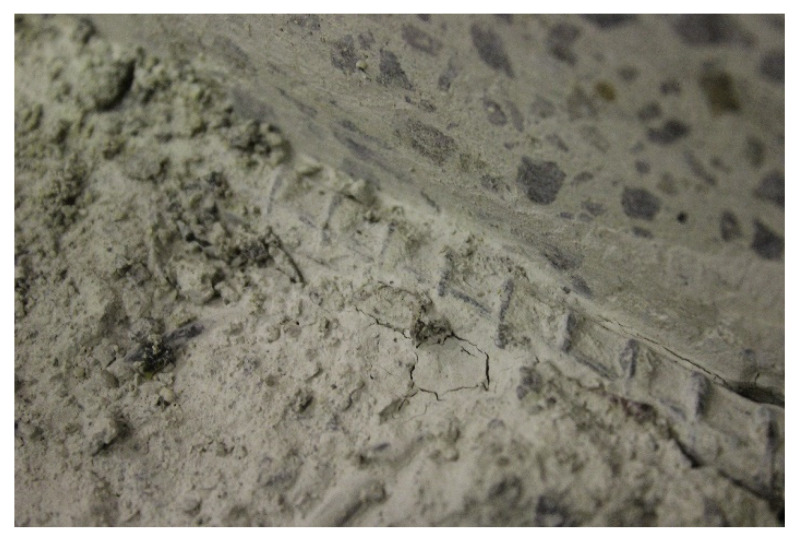
Steel–concrete interface on the OPC beam, showing cracks along the ribs and crushed concrete between ribs.

Figure 11, on the contrary, showed a very different morphology at the OPC–steel interface.

It can be seen that the concrete in front of the ribs was crushed and the ribs were totally exposed after the pull out tests. The cracks spread out from the ribs in different directions, causing the dendritic cracks on the surface of the OPC beams.

#### 3.1.3. Micro-Morphology on the Steel–Concrete Interface (SEM Observation)

**Figure 12:**
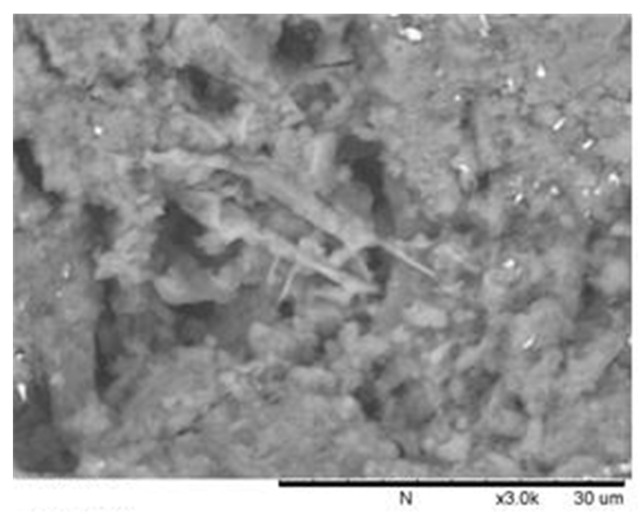
SEM image of a fractured OPC piece from parts adjacent to steel, showing needle-like crystal structures.

After the pull out test, small pieces of concrete were immediately collected from the bond area of the test pieces and SEM observation was performed. Figure 12 illustrates an SEM image of a piece taken from the OPC reinforced concrete interface. From this figure, several crystalline phases were observed. The formation of these crystalline phases results in a much higher than average porosity, which can reduce its intensity in this area. The weak interface area of OPC can explain the concrete crushing and interfacial de-bonding shown in Figure 11.

**Figure 13:**
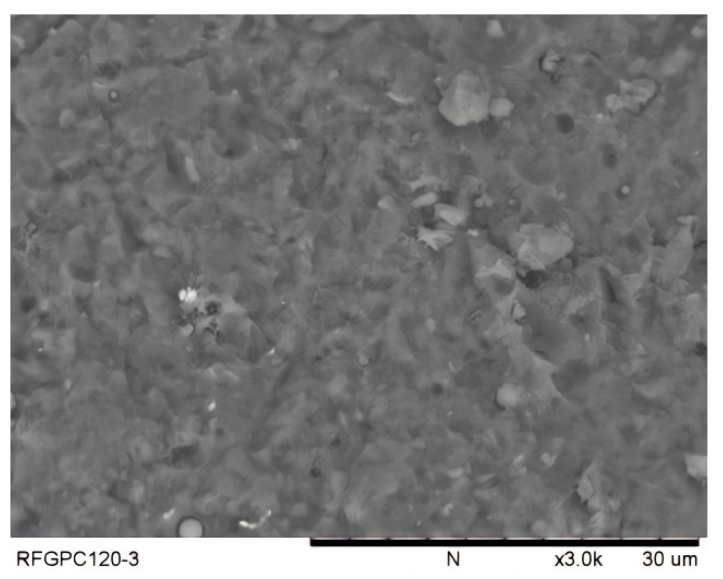
SEM image of fractured GPC pieces from adjacent parts of steel illustrating dense structure.

However, no specific crystals were observed in GPC that could form a weak layer in the interface area. Figure 13 shows that the bond interface of reinforced GPC is more homogenous than the bond interface of OPC concrete. 

The relatively homogeneous micro-morphology of the interface area of reinforced GPC illustrates the bond stiffness shown in Figure 10 and the strong bond strength of plain bar reinforced GPC. For samples reinforced with plain bars, the homogeneous layer between the geopolymer binder and the steel gave a larger contact area than between the highly porous OPC and the steel. This provides high bond strength with a plain bar. For ribbed bars, the homogeneous bond interface of the GPC results in a good load-bearing condition between the rib and the concrete in front of it, causing a high gradient at the elastic stage of the bond–slip curve.

### 3.2. Uniform Bond Strengths

Usually, the bond stress (τ_u_) is assumed to be uniform along the embedded length [45]. According to this assumption, at any moment of loading, the bond stress could be determined by simply dividing the load by the bond area of the bar. The ultimate pull out load is the maximum reading before failure occurs. The bond strength was then derived by dividing the ultimate load by the bond area. By using this approach, the bond stress was calculated and is listed in Table 4. Each load or stress value presented in the table is the mean of three test results.

Table 4 shows the results of experimental and statistical tests. Hypothesis testing has been introduced to identify differences between the test scores of each group. For statistical inference of observed data, the T-test helps to compare whether the means of the two groups differ significantly.

First, the averages of gauged and non-gauged specimens made with the same mixture are compared. Statistical results showed the absence of a significant difference between the gauged and non-gauged samples. Therefore, the results obtained from the gauged and non-measured groups can be considered to belong to the same population compared to other types of reinforced concrete.

Second, the null hypothesis is that the population means of plain bar reinforced OPC and GPC are the same. The null hypothesis is rejected because the absolute value of the test statistic obtained with the plain bar reinforced GPC and OPC of 6.30 is greater than the 95% confidence critical t-value of 2.65, thus it can be concluded the two population means are different at the 0.05 significance level. The *P*-value for this test is 0.0004 (<0.05), which generally means 99.96% statistical confidence. Consequently, it is due to the nature of these two different concretes rather than any other reasons, the two sets of plain bar reinforcement test pieces show different bond stresses. As shown earlier, the magnitude of the bond strength of a sample reinforced with a plain bar mainly depends on the quality of adhesion. The homogeneous morphology of the GPC steel surface shown by SEM is believed to be the cause of the excellent adhesion between the plain bar and GPC.

Following the same procedure to test the mean values of ribbed GPC and OPC with F-test and T-test. The results of the T-test supported the null hypothesis. It gave the solid ground to believe the absence of a significant difference between the mean values of ribbed GPC and ribbed OPC. As illustrated before, the magnitude of the bond strength of the ribbed specimen depends on the magnitude of the mechanical interlock between concrete and steel. Since the specifications of the specimens embedded in these two types of concrete and the ribbed bar are the same, the tensile strength of the surrounding concrete determines the interlocking property. Another paper [46] found that the GPC and OPC blends used in this study had similar tensile strengths. Therefore, there is no significant difference in the bond strength between ribbed GPC and OPC.

### 3.3. Bond–Slip Curves of Beam End Specimens

#### 3.3.1. Plain Bar

Plain bar pull out tests are very important in studying and comparing the chemical adhesion between steel bars and different concretes.

##### Bond–Slip Curves of Plain Bar Reinforced GPC

**Figure 14:**
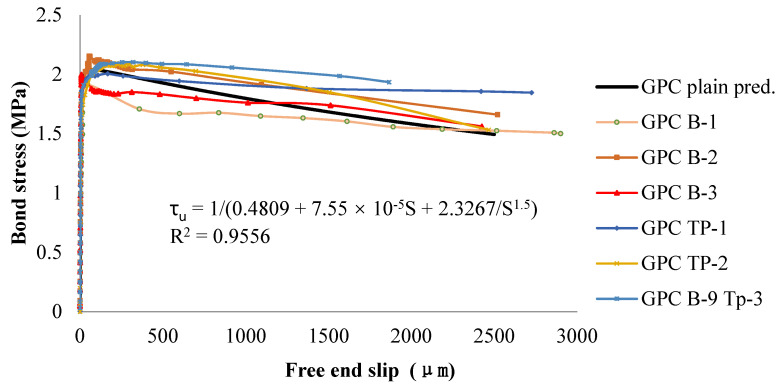
Bond–slip graphs of plain bar reinforced GPC.

Figure 14 is a typical graph of the fundamental relationship in the form of bond stress versus slip for the 16 mm plain round bar reinforced GPC. Each line represents the result obtained for a gauged (TP-x, e.g., GPC TP-1) or a non-gauged (B-x, e.g., GPC B-1) bar reinforced GPC beam. Due to the large number of data points collected, in the ascending part, a 0.5 kN load interval was adopted, and the value of slip was taken at each load interval, while in the sharp descending section, the original data logging rate was followed in the plot. These reversed ‘L’ shape curves describing the fundamental bond stress vs. slip relationship first showed a linear increase in bond stress in the early stages of not yet visible slip. In this part, adhesion was the main contributor to bonding.

Later, the bond–slip relationship changed to nonlinear following increases in the slip of the bar. Once relative slip started, the adhesion between the concrete and steel was quickly lost. As the bar continued to slip, its surface asperities changed with different points of the surrounding concrete, and the bond stress continued to increase, though at a decreasing rate. In this part, friction is the main component of the bond. After reaching a peak, the bond stress starts to decrease gradually with significantly accelerated slip until pull out failure occurs. The descending part of the bond stress would be expected due to the reduction in the confining pressure of the surrounding concrete and the subsequent decrease in the friction force.

##### Bond–Slip Relationship of Plain Bar Reinforced GPC

The prediction was developed for the bond–slip relationship of plain bar reinforced GPC as follows:

(1) $$\tau_{u}=\frac{1}{0.4809+7.55\times 10^{-5\;}S+\frac{2.3267}{s^{1.5}}}$$

where τ_u_ = bond stress in MPa, and S = slip in µm. The square of the correlation coefficient [47] of Equation (1) was considerably high, R^2^ = 0.9556, which indicated that this equation agrees well with the bond–slip relationship observed in experimental tests. The iteration algorithms utilized in the regression were the Levenberg–Marquardt [48] and the general global optimization methods [49]. Regression and optimization were conducted using the math package 1stOPT^®^.

##### Comparison with Plain Bar Reinforced OPC

**Figure 15:**
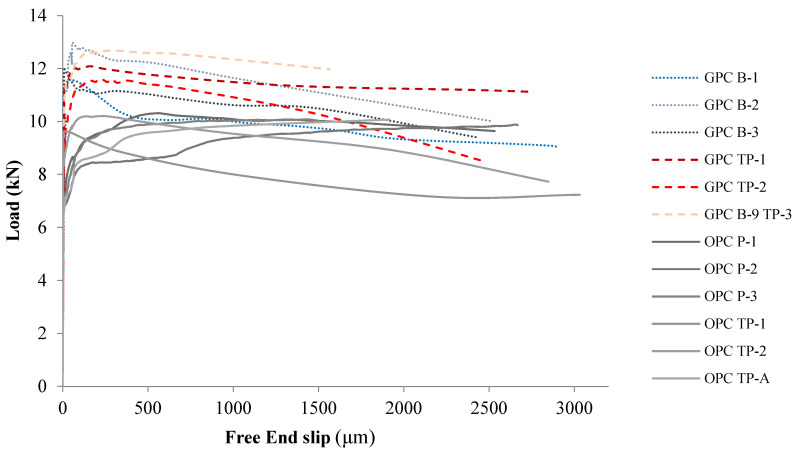
Load–slip graphs of plain bar reinforced OPC and GPC.

Figure 15 exhibits the load–slip graphs of the plain bar reinforced GPC and OPC. The results from the OPC control group were added and each line represents the result obtained for a gauged (P-x, e.g., OPC P-1) or non-gauged (TP-x, e.g., OPC B-1) bar reinforced OPC beam. Initially, the bond load–slip curves of GPC and OPC are both very steep because of adhesion. Once the adhesion force is incapable of resisting the pull out load, the curve starts to separate from the vertical axis quickly. Therefore, the value of the load recorded at the moment of quick separation could be regarded as the magnitude of the maximum adhesion force. In most cases, separation speeded up after the curve reached the peak value. The height of the inflexion points on the load–slip graphs thus represents the magnitude of the adhesion force between the steel and concrete. It is clear that the inflexion points in plain GPC samples are much higher than those in plain OPC, meaning that GPC samples have stronger adhesion with plain steel bars than OPC. The bond strength between GPC and steel bars is on average 21% higher than the bond strength between OPC and plain bars.

It is not surprising to observe a large plain bar pull out load on the GPC specimens. First, strong adhesion between GPC and steel has been noticed even before any mechanical tests were performed. For casting, GPC specimens must be poured into plastic moulds because the adhesion between GPC and the surface of steel moulds is so strong that it is impossible to remove the GPC samples from the steel moulds after hardening. This strong adhesion with steel causes GPC to experience a higher bond stress than OPC. In addition, from the SEM images, it has been shown that the contact surface between steel and GPC is more homogenous than that between OPC and steel. A larger contact area also contributes to the difference in adhesion capability between them.

#### 3.3.2. Ribbed Bar

##### Bond–Slip Curves of Ribbed Bar Reinforced GPC and OPC

**Figure 16:**
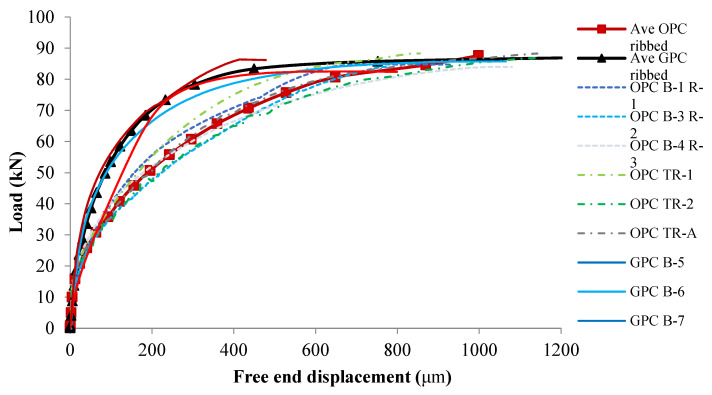
Load–slip graphs of ribbed bar reinforced OPC and GPC.

Figure 16 plots the six ribbed GPC and six ribbed OPC experimental load–slip curves and the average analytical bond–slip curve of each concrete. Although the ribbed bar reinforced pull out samples all failed by sudden splitting, long slippages were observed for each sample before failure. Due to the large number of data points collected, while plotting the average curves, a 5 kN load interval was adopted, and the value of slip was taken at each load interval [50]. The average slip value at a particular load for all the bars of one group was then calculated.

It has been proven that there is no significant difference between the mean values of the bond strength of ribbed GPC and OPC. However, it was observed in Figure 14 that the patterns of the bond–slip curves of different concretes are very different. The curves of ribbed bar reinforced GPC have a dramatically higher ascending branch than those of OPC. At the same load, OPC exhibits larger slip values than GPC. As discussed in Section 3.1.3, this is caused by the homogenous interfacial area between the GPC and steel bars. After reaching 20 kN, the load increasing rate decreased sharply in the OPC curves but gently in the GPC curves, so the analytical bond–slip curves of OPC and GPC separated from each other at this point. After that, the load in the GPC samples kept rapidly increasing until the slip value reached 200 microns. After the load was applied, the load increasing rate in the GPC specimen slowed and stopped at approximately 420 microns, with dramatically accelerated slip development. The load in the OPC samples continued to increase until failure occurred. The maximum point of each ribbed OPC curve always occurred at, or very close to, the end of the loading procedure.

##### Energy Consumption during the Pull out Test of Ribbed Bar Reinforced GPC and OPC

Even though the ribbed GPC and OPC have almost equivalent bond strengths, the areas under their load–slip curves are different, and consequently, their energy absorption profiles are different.

(2) $$\mathrm{WGPC}\;=\int_{0}^{1000}f\;\left(s\right)=\int_{0}^{1000}-3.697-0.006\;S^{1.5}+0.000026\;S^{2.5}+6.268S^{0.5}=0.0763$$

(3) $$\mathrm{WOPC}\;=\int_{0}^{1000}f\;\left(s\right)=\int_{0}^{1000}-1.111-0.158\;S+0.006\;S^{1.5}-0.00011\;S^{2}+4.992\;S^{0.5}=0.0678$$

Integrating the bond–slip curves of ribbed GPC from 0 to 1000 microns gives 0.0763 kN·m, while integrating the curves of ribbed OPC from 0 to 1000 microns gives 0.0678 kN·m, which is 11.1% less than that of the GPC samples. This indicates that in the pull out procedure, more energy was absorbed by the GPC samples. More work (E, kN·m) is needed to break the bond between the GPC and steel bars.

##### Bond–Slip Relationship of Ribbed Reinforced GPC

The bond–slip curves of ribbed bar reinforced GPC and OPC specimens are plotted in Figure 15, where each line represents the average result obtained for a group of six identical samples.

**Figure 17:**
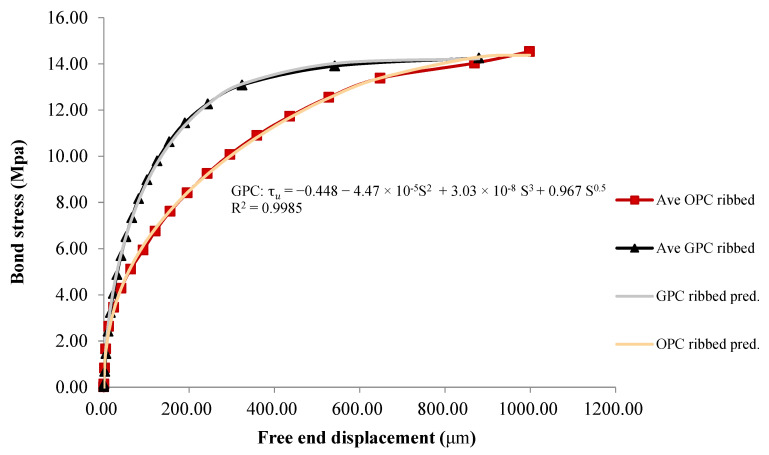
Statistical regression model of the bond–slip relationship between the ribbed bar and GPC.

Prediction models have been developed for the bond–slip relation of ribbed bar reinforced GPC, and the analytical curve is plotted in Figure 17.

(4) $$\tau_{u}=-0.448-4.47\times 10^{-5}{\;S}^{2}+3.03\times 10^{-8}{\;S}^{3}+0.967{\;S}^{0.5}$$

where τ_u_ = bond stress in MPa, and S = slip in microns. The R^2^ value of this model is 0.9985. The regression model derived from the ribbed GPC bond–slip relationship is more accurate than that derived from the plain bar tests, which can be attributed to the low variability of the data set achieved in ribbed bar pull out tests.

The iteration algorithm utilized in the regression was the Levenberg–Marquardt algorithm, and the general global optimization method was also used for model optimization. Regression and optimization were conducted with the math package 1stOPT^®^.

##### Comparison of Ribbed Bar Reinforced GPC and OPC

To statistically examine the difference in the GPC and OPC concrete relationships, a paired sample *t*-test is introduced. This is a statistical technique used to compare two population means in the case of two correlated samples. Compared with the standard *t*-test, which compares two groups of samples that are independent of each other, the paired sample *t*-test can determine whether there is a significant difference between the mean values of the correlated ‘measurement’ under two different ‘conditions’ [51]. Specifically, the ‘measurement’ here is the slip values at each bond stress level, and the ‘conditions’ are the two kinds of reinforced concrete, as shown in Table 5.

If we assume that the difference between the slip values of GPC and OPC concrete at a specific bond stress is ΔXi, the null hypothesis is that for a specific value of bond stress, any difference is due to meaningless chance. In contrast, the study hypothesis is as follows: for a specific value of bond stress, any difference is due to the material differences between these two concretes.

Since t_stat_ = 4.325 > t_0.05_ = 2.093 and *P* = 0.000365 < 0.05 = α, we reject the null hypothesis and conclude with 95% confidence that the difference between the bond stresses of GPC and OPC concrete at the same slip value was not due solely to chance. There was a statistically significant difference between the bond–slip curves of ribbed GPC and OPC concrete. For this reason, it would be inappropriate to use the empirical equations derived to describe the bond–slip curves of reinforced OPC concrete to also express those of the corresponding GPC concrete.

The consolidated microstructure of GPC endows it with the ability to stop or delay the development of cracks and maintain adequate bond stress with steel. When the ribbed bar reinforced GPC was subjected to the same magnitude of pull out load as OPC, the relative slip between GPC and the steel bar was smaller than that between OPC and the steel bar, which indicates that GPC possesses a higher ‘bond stiffness’ than OPC. In addition, it is expected to have high stiffness due to the high adhesion between steel and the dense interface structure of GPC.

## 4. Conclusions

This study investigates the bond performance of geopolymer concrete to steel reinforcing bars. ASTM A944 beam end tests were conducted on 12 GPC and 12 OPC beams. It has been proven by statistical testing that GPC behaves differently from OPC in terms of bond behaviour. The following conclusions are drawn from the experimental study and statistical testing:

(1) The strong adhesion between the geopolymer binder and steel and the homogenous interfacial structure of the reinforced GPC contribute to the approximately 21% higher bond strength achieved with the plain bars.

(2) The paired T-tests performed for the bond–slip curves of GPC and OPC proved the significantly high bond stiffness of GPC. The bond stress plotted for ribbed OPC at any given value of slip in the ascending branch was less than that plotted for ribbed GPC. The initially ascending branch of plain bar reinforced GPC grows longer than that of plain OPC.

(3) The statistical tests proved that GPC and OPC possess different bond behaviours with steel bars. Specifically, plain bar reinforced GPC has a higher mean value than plain bar reinforced OPC, and ribbed bar reinforced GPC has a higher energy absorption ability than ribbed bar reinforced OPC. Conclusively, GPC has a better bond performance than OPC, which is suited to be used in steel bar reinforced components.

(4) Reinforced geopolymer concrete with plain bars and ribbed bars are all based on the fact that they behave differently from reinforced OPCs in terms of bond–slip. The enhanced GPC bond–slip model was presented according to a statistical regression of experimental results.

The experimental results of this study demonstrated that there were significant differences in the bond behaviour between GPC and OPC. Moreover, the microchemical and physical structures that contributed to these differences are the focus of the authors’ next research. The authors are working on the representation and visualization of the interfacial transition zone (ITZ) between geopolymer and reinforcements to provide an informed explanation of the behaviour of microcomposites in geopolymer concrete.

## Figures and Tables

**Table 1:** Chemical composition of the fly ash as determined by XRF (quantitative results).

Fly Ash Batches	CFA 1	CFA 2
component	wt%	wt%
SiO_2_	58.491	57.360
Al_2_O_3_	21.046	22.106
Fe_2_O_3_	8.286	8.126
CaO	3.843	4.701
K_2_O	3.938	3.090
TiO_2_	2.232	2.445
SO_3_	1.282	1.098
SrO	0.340	0.489
ZrO_2_	0.226	0.263
MnO	0.158	0.189
Rb_2_O	0.045	0.053
Y_2_O_3_	0.032	0.043
LOI [36]	1.6	0.91
SiO_2_/Al_2_O_3_ (wt)	2.78	2.59

**Table 2:** Mix proportion of concrete (kg/m^3^).

Ingredients	GPC	OPC
14 mm aggregate	500	242
10 mm aggregate	310	353
7 mm aggregate	280	349
River sand	630	814
Class F Fly Ash	420	-
Cement	-	357
12 mol/L NaOH	60	-
Na_2_SiO_3_	150	-
Water	31	225
MWR	4	-
VM	4	-

**Table 3:** Properties of test bars.

Steel Bars	Diameter, mm	Nominal Area, mm^2^	Yield Strength, MPa	Ultimate Strength, MPa
Ribbed bar	16	201	546	633
Plain bar	16	201	339	507

**Table 4:** Bond strength of OPC and GPC specimens.

Group ID		Ultimate Load, P (KN)	Uniform Bond Stress, τ_u_ (MPa)	Standard Deviations	*t*-Test Result of Mean of Gauged and Non-Gauged	*t*-Test Result of Mean of GPC and OPC
GPC plain	GPC plain gauged	12.19	2.02	0.12	*p* = 0.57 > 0.05, no significant difference from the reference	t = 6.30 > 2.65, *p* = 0.0004 < 0.05, significant difference from
	GPC plain non-gauged	12.46	2.01	0.05	Reference	OPC plain
GPC ribbed	GPC ribbed gauged	87.16	14.46	0.21	*p* =0.17 > 0.05, no significant difference from the reference	t = 0.39 < 2.23, *p* = 0.35 > 0.05, no significant difference from
	GPC ribbed non-gauged	84.73	14.06	0.33	Reference	OPC ribbed
OPC plain	OPC plain gauged	10.09	1.67	0.04	*p* = 0.95 > 0.05, no significant difference from the reference	Reference
	OPC plain non-gauged	10.09	1.68	0.07	Reference	
GPC ribbed	GPC ribbed gauged	84.27	13.98	0.08	*p* = 0.12 > 0.05, no significant difference from the reference	Reference
	GPC ribbed non-gauged	86.79	14.10	0.26	Reference	

**Table 5:** The slip values and corresponding bond stresses.

Bond Stress, τ_u_ (MPa)	Slip, S (µm)	
	GPC	OPC Concrete
0.025	0	0.01
0.14	0.28	0.56
0.76	2.23	2.20
1.58	5.32	4.76
2.525	10.05	11.74
3.35	15.50	23.43
4.18	22.34	40.51
5.00	30.80	63.21
5.82	40.48	92.46
6.64	51.98	120.73
7.475	65.70	155.70
8.29	81.80	195.72
9.12	101.36	242.71
9.94	124.86	296.09
10.77	153.43	359.1
11.59	190.05	436.21
12.41	243.63	527.84
13.24	324.16	648.05
13.97	541.40	869.45
14.39	879.15	998.28

## Data Availability

The data presented in this study are available on request from the corresponding author.
